# Optimal Pharmacologic Treatment of Heart Failure With Preserved and Mildly Reduced Ejection Fraction: A Meta-analysis

**DOI:** 10.1001/jamanetworkopen.2022.31963

**Published:** 2022-09-20

**Authors:** Boyang Xiang, Ruiqi Zhang, Xiaoguang Wu, Xiang Zhou

**Affiliations:** 1 Department of Cardiology, The Second Affiliated Hospital of Soochow University, Suzhou, China

## Abstract

**Importance:**

In recent years, significant progress has been made in the pharmacologic treatment of heart failure (HF) with reduced ejection fraction (HFrEF), but there is still insufficient evidence for drug therapy for HF with preserved ejection fraction (HFpEF) and mildly reduced ejection fraction (HFmrEF).

**Objective:**

To compare the outcomes associated with different drug combinations for the treatment of HFpEF and HFmrEF.

**Data Sources:**

A search of the PubMed, Embase, and Cochrane Central Register of Controlled Trials (CENTRAL) databases was conducted for studies published from inception to October 9, 2021.

**Study Selection:**

Randomized clinical trials on the use of angiotensin-converting enzyme (ACE) inhibitors, angiotensin receptor blockers (ARBs), angiotensin receptor-neprilysin inhibitors (ARNIs), mineralocorticoid receptor antagonists (MRAs), β-blockers, and sodium-glucose cotransporter 2 (SGLT2) inhibitors for patients with HFpEF or HFmrEF.

**Data Extraction and Synthesis:**

Data extraction and bias assessment were independently performed by 2 reviewers following the Preferred Reporting Items for Systematic Reviews and Meta-analyses (PRISMA) guideline. All data for 3 outcomes were pooled with a fixed-effect model.

**Main Outcomes and Measures:**

The main outcomes were first hospitalization for HF, all-cause mortality, and cardiovascular mortality. Hazard ratios (HRs) and 95% credible intervals (CrIs) were evaluated using a bayesian network meta-analysis model.

**Results:**

In this analysis, 19 randomized clinical trials, including 20 633 patients with HF and an ejection fraction of 40% or more, without a remarkable risk of bias were included. Compared with placebo, no treatments were associated with a significant reduction in the risk of all-cause death or cardiovascular death. SGLT2 inhibitors, ARNIs, and MRAs were associated with a significant decrease in the risk of HF hospitalization compared with placebo (SGLT2 inhibitors: HR, 0.71 [95% CrI, 0.60-0.83]; ARNIs: HR, 0.76 [95% CrI, 0.61-0.95]; MRAs: HR, 0.83 [95% CrI, 0.69-0.99]), and SGLT2 inhibitors were the optimal drug class in terms of reducing the risk for HF admission. Sensitivity analysis results demonstrated a progressive decrease in the risk of HF admission and an advance in mean rank associated with the increasing use of drug classes.

**Conclusions and Relevance:**

The findings of this study suggest that SGLT2 inhibitors were the optimal drug class for HFpEF and HFmrEF, consistent with the most recent guideline recommendation. The incremental use of combinations of SGLT2 inhibitors, ACE inhibitors or ARBs, and β-blockers may be associated with accumulative benefits in HF hospitalization rather than all-cause death among patients with HFpEF and HFmrEF.

## Key Points

**Question:**

What is the optimal drug combination for treatment of heart failure (HF) with preserved ejection fraction (HFpEF) and mildly reduced ejection fraction (HFmrEF)?

**Findings:**

In this network meta-analysis, 19 randomized clinical trials enrolling 20 633 patients with HFpEF or HFmrEF were included. Compared with placebo, no included drug classes were associated with a reduced risk of death, but sodium-glucose cotransporter 2 (SGLT2) inhibitors, angiotensin receptor-neprilysin inhibitors, and mineralocorticoid receptor antagonists were associated with a significant decrease in hospital admission for HF; SGLT2 inhibitors were the optimal drug class for decreasing the risk of admission for HF.

**Meaning:**

The results of this study suggest that the incremental use of combinations of SGLT2 inhibitors, angiotensin-converting enzyme inhibitors–angiotensin receptor blockers, and β-blockers was associated with accumulative benefits in HF hospitalization rather than all-cause death among patients with HFpEF and HFmrEF.

## Teaser

This network meta-analysis compares the effectiveness of different drug combinations for the treatment of heart failure with preserved ejection fraction and heart failure with mildly reduced ejection fraction.

## Introduction

Approximately 64 million people worldwide have heart failure (HF), with considerable related morbidity and mortality.^1,2^ Based on the left ventricular ejection fraction (LVEF), recent guidelines classify HF into HF with reduced ejection fraction (HFrEF), HF with mildly reduced ejection fraction (HFmrEF), and HF with preserved ejection fraction (HFpEF).^3,4^

For 30 years, pharmacologic therapy for HFrEF has continued to improve. Sodium-glucose cotransporter 2 (SGLT2) inhibitors, mineralocorticoid receptor antagonists (MRAs), angiotensin receptor blockers (ARBs), angiotensin-converting enzyme (ACE) inhibitors, β-blockers, and angiotensin receptor-neprilysin inhibitors (ARNIs) have been recommended as disease-modifying treatments for patients with HFrEF by US and European clinical guidelines.^3,4^ The incremental use of combinations of these drugs has contributed to progressive benefits for patients with HFrEF.^5^

Nevertheless, the development of medical therapy for HF with higher ejection fraction has failed to meet expectations. Many large-scale clinical trials on ARNIs, MRAs, ACE inhibitors, ARBs, and β-blockers demonstrated neutral results in the primary composite outcome of death or HF hospitalization for patients with HFpEF and HFmrEF.^6^ Still, these drug classes have been recommended to treat HFpEF and HFmrEF by US guidelines because of their marginal benefits regarding HF hospitalization.^4^ Recently, a large clinical trial found that, for patients with HFpEF and HFmrEF, empagliflozin significantly improved the primary composite end point of HF hospitalization or cardiovascular death compared with placebo, which was attributed to a decrease in HF hospitalizations without a significant decrease in mortality.^7^ Therefore, SGLT2 inhibitors have been recommended for patients with HFpEF or HFmrEF in preference to other drug classes in the latest US guideline.^4^ To our knowledge, there have been no head-to-head studies comparing SGLT2 inhibitors with other HF drug classes for this population.

Network meta-analysis is a reliable method to simultaneously compare multiple different interventions without head-to-head studies. In this article, we used a bayesian network meta-analysis to compare the outcomes associated with these HF treatment drugs for patients with HFpEF or HFmrEF and to explore whether the benefits associated with these drugs for patients with HF with higher ejection fraction were as accumulative as for patients with HFrEF.^5^

## Methods

### Search Strategies and Selection Criteria

This study was conducted following the Preferred Reporting Items for Systematic Reviews and Meta-analyses (PRISMA) reporting guideline and extension statement for reporting of network meta-analysis.^8^ We searched the PubMed, Embase, and Cochrane Central Register of Controlled Trials (CENTRAL) databases from inception to October 9, 2021, using strategies presented in the eAppendix in the Supplement. This analysis included randomized clinical trials (RCTs) with follow-up of more than 3 months of adult patients with symptomatic HF and LVEF of 40% or more, assessing any of the following interventions: ARNIs, MRAs, ACE inhibitors, ARBs, SGLT2 inhibitors, and β-blockers.

An RCT was excluded if the whole trial population had the following characteristics that possibly affected treatment response: (1) same sex, (2) comorbidity (eg, hypertension, diabetes, or atrial fibrillation), (3) ischemic HF, (4) acute HF, or (5) myocardial infarction. Trials including only a proportion of patients with these characteristics were included. Cardiac function is known to be significantly associated with the outcomes of patients with HF; therefore, trials enrolling mostly patients with severe cardiac function were also removed because of the homogeneity of the evidence.

### Study Selection and Data Extraction

Study selection, bias assessment, and data extraction were conducted independently by 2 researchers (B.X. and R.Z.), and divergences were resolved by discussion. For all eligible studies, data on study design, population characteristics, interventions, and outcomes were extracted into a database. The risk of bias for eligible studies was assessed across 7 domains.^9^

### Outcome Measures

Outcomes estimated in this analysis included all-cause death, cardiovascular death, and first hospital admission for HF during the entire follow-up period. These outcomes were reported as efficacy or safety end points in the included studies.

### Statistical Analysis

Based on the combination of direct and indirect study evidence, bayesian network meta-analysis can indirectly compare the outcomes associated with different interventions when direct evidence is absent. In the present study, previously described bayesian network meta-analysis models were applied.^10^ These models combined binary and time-dependent data to ensure the integrity of the data while preserving their time attributes. Time-dependent data were preferentially entered into the model to reduce the loss of their time attributes. Unless a higher between-study heterogeneity was found or the random-effect model was more parsimonious than the fixed-effect model, priority was given to showing results from the fixed-effect model for easier interpretation of analysis results. Noninformative prior distributions were used: normal distributions (mean, 0; variance, 10^6^). We used OpenBUGS, version 3.2.3 (University of Cambridge) to conduct network analysis with published codes based on 200 000 iterations with 2 chains and a 100 000 burn-in period. The posterior distributions of the model were presented as hazard ratios (HRs) and 95% credible intervals (95% CrIs). The probability (*P* score) that the therapy was associated with better outcomes than the comparator was presented after the transformation of the estimated points and their 95% CrIs. Heterogeneity was assessed with the *I*^2^ statistic, and inconsistency was assessed with the node-split model using R, version 4.1.1 (R Group for Statistical Computing).^11^ If there were closed loops in the network plot, the *P* value of inconsistency could be calculated; otherwise, the *P* value was not available. A 2-sided *P* < .05 was considered statistically significant. Ranking probabilities were presented in the form of a rankogram plot.

To weaken the interference of concomitant drugs with analysis results, a prespecified sensitivity analysis was performed. If more than 50% of the patients in a trial were using the drug of interest at baseline, intervention classes were regarded as including the concomitant drug. The threshold defining the concomitant therapy referred to published articles.^5,12,13^ When more than 50% of patients in a trial received concomitant drugs, the intervention class was classified as a combined treatment (primary trial drug class plus concomitant drug class). If necessary, additional sensitivity analysis was performed.

## Results

### Study Selection and Characteristics

**Figure 1.**
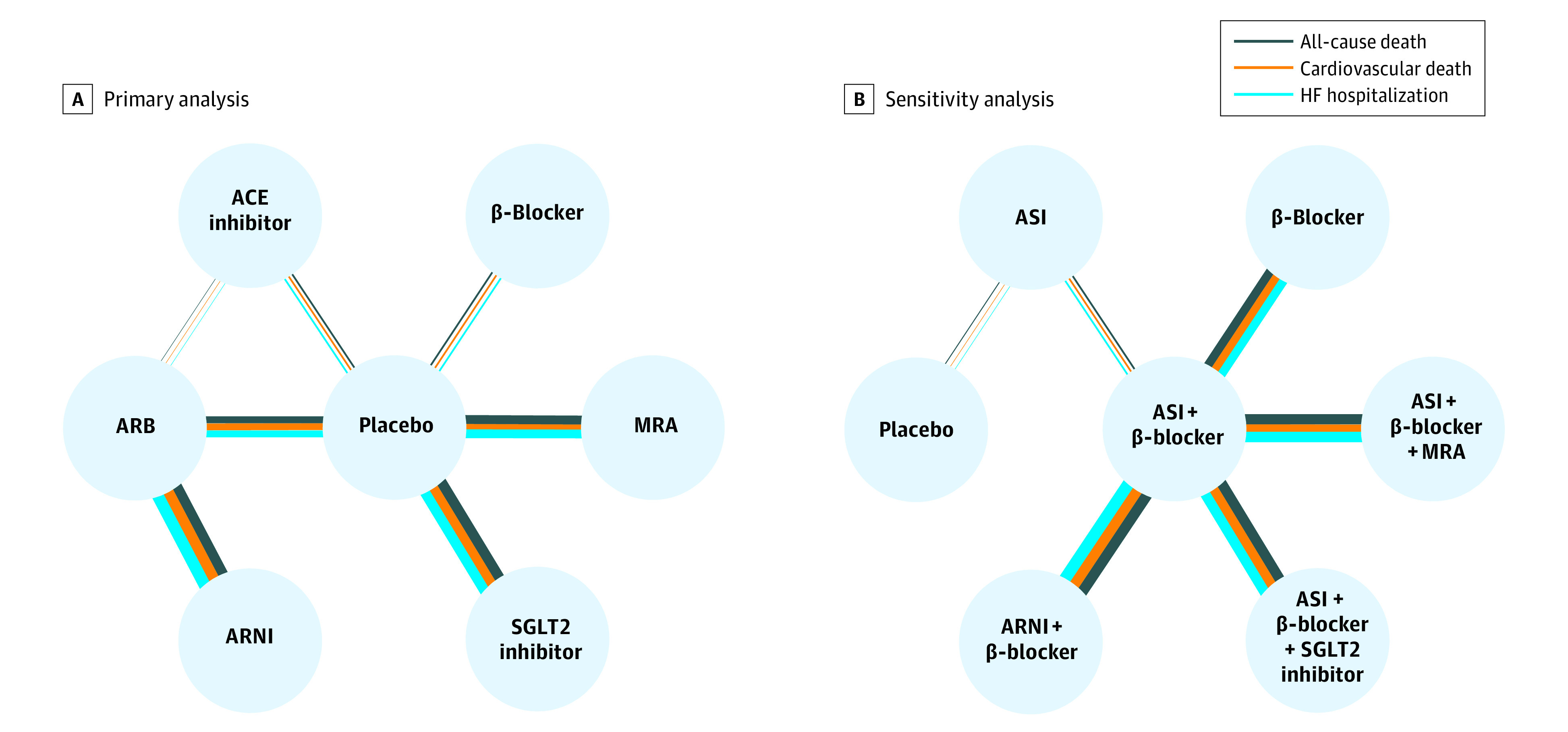
Network Plots of Direct Evidence The width of the connecting line was positively correlated with the patient-years of direct evidence. Different colors of connecting lines corresponded to different outcomes. ACE indicates angiotensin-converting enzyme; ARB, angiotensin receptor blocker; ARNI, angiotensin receptor-neprilysin inhibitor; ASI, angiotensin system inhibitor (ACE inhibitor or ARB); HF, heart failure; MRA, mineralocorticoid receptor antagonist; and SGLT2, sodium-glucose cotransporter 2.

In this network analysis, 11 855 publications were identified, and 19 RCTs, including a total of 20 633 patients with HF and an ejection fraction of 40% or more, were included after screening (eFigure 1 in the Supplement); 19 trials reported all-cause death, 16 trials reported cardiovascular death, and 12 trials reported hospital admission for HF. Network diagrams of direct evidence based on RCTs are presented in Figure 1. Multiple direct connections among the intervention classes comprised 52 761 patient-years for death from any cause, 52 090 patient-years for death from cardiovascular causes, and 50 524 patient-years for HF hospitalization.

Most of the included studies were double-blind, multicenter RCTs, and nearly all of the included RCTs had no significant risk of bias (eFigure 2 in the Supplement). The median trial duration ranged from 4 to 40 months, with the median duration of only 1 study being less than 6 months. The sample sizes of the included trials ranged from 40 to 5988 patients, with 6 trials enrolling fewer than 100 patients and 6 trials enrolling more than 500 patients.

**Figure 2.**
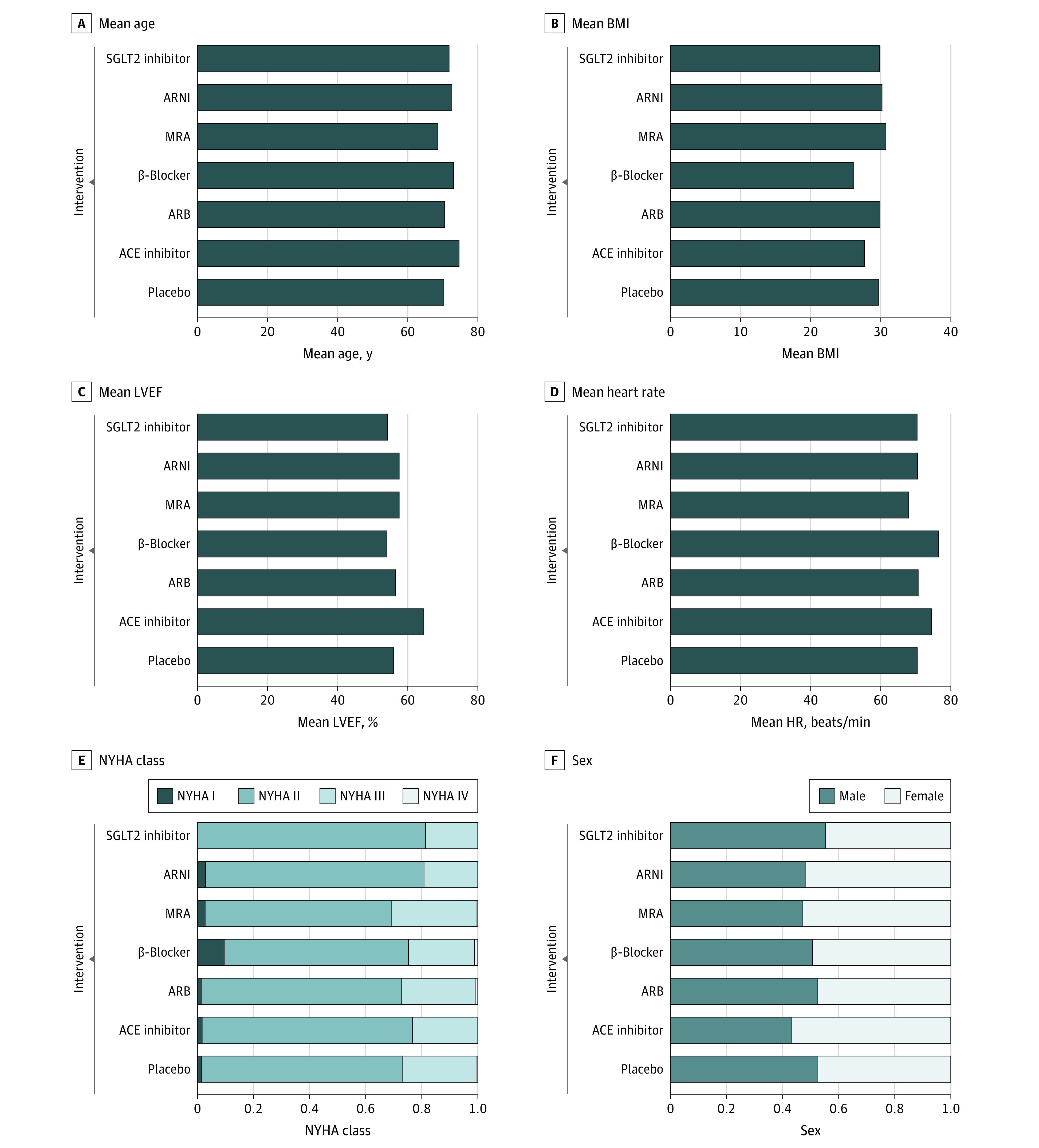
Study Population Characteristics at Baseline Population characteristics at baseline were stratified by 7 intervention classes (6 medication classes plus placebo) and were overall deemed to be similar among the intervention classes. ACE indicates angiotensin-converting enzyme; ARB, angiotensin receptor blocker; ARNI, angiotensin receptor-neprilysin inhibitor; BMI, body mass index (calculated as weight in kilograms divided by height in meters squared); HR, heart rate; LVEF, left ventricular ejection fraction; MRA, mineralocorticoid receptor antagonist; NYHA, New York Heart Association; and SGLT2, sodium-glucose cotransporter 2.

Figure 2 shows the population characteristics at baseline stratified by intervention classes, demonstrating the transitivity of our network meta-analysis. The age, sex, obesity degree, hemodynamics, and LVEF of the populations were deemed similar among the 7 different intervention classes (6 medication classes plus placebo). The cardiac function of the eligible population was primarily concentrated in New York Heart Association Class II and III. More detailed information about study characteristics is presented in eTable 1 in the Supplement. The background therapies of the included patients are presented in eTable 2 in the Supplement. The numbers of patients taking ARBs and ACE inhibitors at baseline were not reported separately in many studies; thus, the 2 classes of drugs together were classified as angiotensin system inhibitors (ASIs) in the sensitivity analysis.

### Primary Network Meta-analysis

**Figure 3.**
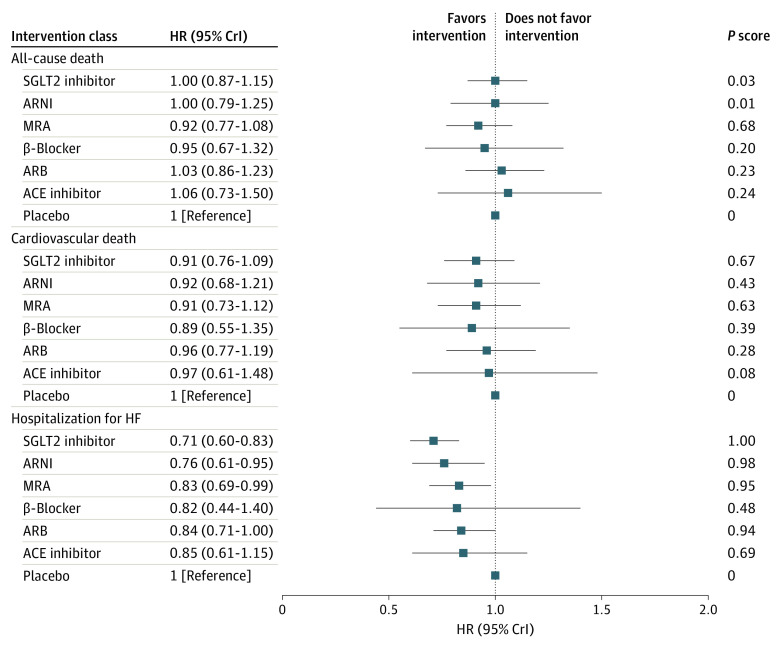
Effect Sizes of Interventions vs Placebo for 3 Outcomes in the Primary Network Meta-analysis A hazard ratio (HR) less than 1 favors the intervention, and a 95% credible interval (CrI) not including 1 signifies statistical significance. A larger *P* score suggests that an intervention is associated with a better outcome. ACE indicates angiotensin-converting enzyme; ARB, angiotensin receptor blocker; ARNI, angiotensin receptor-neprilysin inhibitor; HF, heart failure; MRA, mineralocorticoid receptor antagonist; and SGLT2, sodium-glucose cotransporter 2.

Based on the combination of direct and indirect evidence from the included RCTs, the comparative effectiveness of 7 intervention classes for 3 outcomes were assessed with a fixed-effect model, and the results of the primary analysis are presented in Figure 3 and eFigure 3 in the Supplement. No significant heterogeneity or inconsistency for the 3 outcomes was found. As shown in Figure 3, 6 treatments were not associated with benefits with respect to the risk of all-cause or cardiovascular death compared with placebo. However, SGLT2 inhibitors, ARNIs, and MRAs were significantly associated with a decreased risk of hospital admission for HF compared with placebo (SGLT2 inhibitors: HR, 0.71 [95% CrI, 0.60-0.83]; ARNIs: HR, 0.76 [95% CrI, 0.61-0.95]; MRAs: HR, 0.83 [95% CrI, 0.69-0.99]). In eFigure 4 in the Supplement, the ranking probabilities for all interventions for 3 outcomes are demonstrated. The rankogram plot for HF hospitalization showed that SGLT2 inhibitors were most likely the optimal treatment, followed by ARNIs and MRAs, which was consistent with the rank of estimated points.

### Sensitivity Analysis

**Figure 4.**
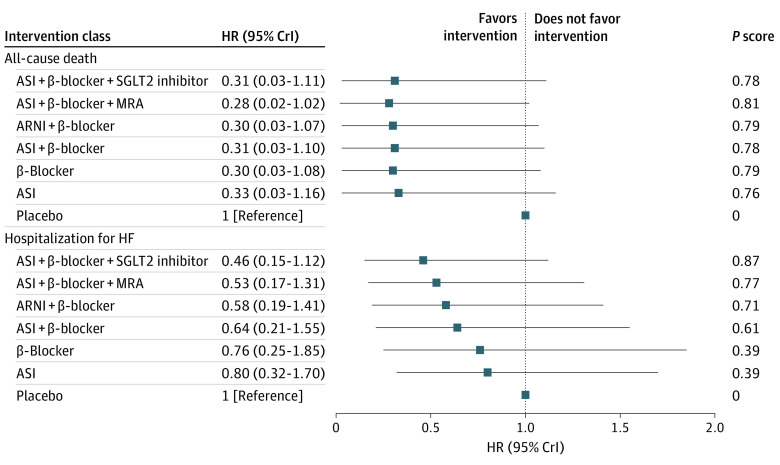
Effect Sizes of Interventions vs Placebo for 2 Outcomes in the Sensitivity Analysis A hazard ratio (HR) less than 1 favors the intervention, and a 95% credible interval (CrI) not including 1 signifies statistical significance. An intervention associated with a better outcome has a larger *P* score. ARNI indicates angiotensin receptor-neprilysin inhibitor; ASI, angiotensin system inhibitor; HF, heart failure; MRA, mineralocorticoid receptor antagonist; and SGLT2, sodium-glucose cotransporter 2.

**Figure 5.**
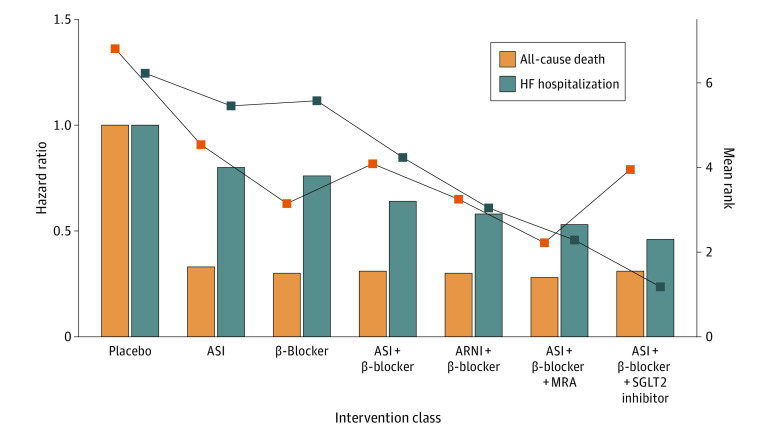
Effect Sizes of Interventions in All-Cause Death and Hospitalization for Heart Failure (HF) The point estimates of interventions compared with placebo in all-cause death and HF hospitalization are summarized with bars. The mean ranks estimated based on the ranking probabilities are summarized with lines. An intervention associated with a better outcome has a smaller mean rank. ARNI indicates angiotensin receptor-neprilysin inhibitor; ASI, angiotensin system inhibitor; MRA, mineralocorticoid receptor antagonist; and SGLT2, sodium-glucose cotransporter 2.

Owing to the poor fit of the model for cardiovascular death, which was possibly associated with the relatively sparse incidence of cardiovascular death and the relatively uneven distribution of direct evidence among each connection, we evaluated the association of 7 intervention classes only with death from any cause and hospitalization for HF with a fixed-effect model. The results of the sensitivity analysis are summarized in Figure 4 and eFigure 5 in the Supplement. No significant heterogeneity was found. In Figure 4, none of the 6 interventions was associated with a significant reduction in all-cause death or HF hospitalization compared with placebo, but the probability that the therapy was associated with greater decrease in HF hospitalization than placebo increased gradually with the incremental use of drug classes. The point estimates and mean ranks of interventions for all-cause death and HF hospitalization are summarized in Figure 5, illustrating a progressive decrease in the risk of HF admission and an advance in the mean ranks of interventions with the increasing use of drug classes. This trend was not observed for all-cause mortality. As shown in eFigure 5 in the Supplement, ASIs plus β-blockers plus SGLT2 inhibitors were associated with a significant reduction in the risk of HF hospitalization compared with ASIs (HR, 0.57 [95% CrI, 0.30-0.98]), β-blockers (HR, 0.60 [95% CrI, 0.48-0.75]), ASIs plus β-blockers (HR, 0.71 [95% CrI, 0.60-0.83]), and ARNIs plus β-blockers (HR, 0.79 [95% CrI, 0.64-0.97]). In addition, ASIs plus β-blockers (HR, 0.85 [95% CrI, 0.72-0.99]), ARNIs plus β-blockers (HR, 0.77 [95% CrI, 0.62-0.94]), and ASIs plus β-blockers plus MRAs (HR, 0.71 [95% CrI, 0.55-0.89]) were associated with a decreased risk of HF admission compared with β-blockers. Moreover, ASIs plus β-blockers plus MRAs were associated with a significant reduction in HF hospitalization compared with ASIs plus β-blockers (HR, 0.83 [95% CrI, 0.69-0.99]). The rankogram plot for HF hospitalization showed that ASIs plus β-blockers plus SGLT2 inhibitors was most likely the optimal therapy, followed by ASIs plus β-blockers plus MRAs and ARNIs plus β-blockers (eFigure 6 in the Supplement). The results of the sensitivity analysis did not conflict with the results of the primary analysis.

Our primary analysis demonstrated positive results only in terms of HF hospitalization. Our network meta-analysis included 6 studies enrolling fewer than 100 patients, of which 4 studies reported hospital admission for HF. Additional sensitivity analysis was performed to ensure the stability of the results of the primary analysis for HF hospitalization. The results of the network meta-analysis for HF hospitalization after removing certain studies with small sample sizes are presented in eTable 3 in the Supplement, illustrating that these studies with small sample sizes had little impact on our results and that our results were relatively stable.

## Discussion

The results of the primary analysis demonstrated that SGLT2 inhibitors, ARNIs, and MRAs were associated with a significant reduction in the risk of HF admission compared with placebo for patients with HFpEF or HFmrEF and that SGLT2 inhibitors were most likely the optimal drug class, in line with the latest guideline. The results of the sensitivity analysis showed that the incremental use of the included drug classes was probably associated with a progressive decrease in the risk of HF hospitalization among patients with HF and an LVEF of 40% or more.

A previous network meta-analysis demonstrated that, for patients with HF and an LVEF of 40% or more, ARNIs and ACE inhibitors were associated with a significant decrease in the risk of hospital admission for HF, but MRAs were not.^14^ These results are partly inconsistent with the results of our analysis. In the previous analysis, a positive result of an ACE inhibitor trial during the first year, rather than a negative result during the entire follow-up, was extracted,^15^ and a result of cardiac hospitalization in an MRA trial was extracted as a result of HF hospitalization.^16^ These differences may have led to the inconsistencies between our analysis and the previous analysis.

In our sensitivity analysis, we focused on the trend of estimated points rather than the estimated points themselves. Although 6 treatments were not significantly associated with a reduction in the risk of hospital admission for HF compared with placebo, which was likely owing to a weak connection between ASIs and placebo, the downward trend of the estimated points for the 6 treatments vs placebo suggested that the increasing use of drug classes was probably associated with the incremental benefits associated with HF hospitalization. Moreover, the probability that the therapy was associated with a greater decrease in HF hospitalization compared with placebo increased gradually with the increasing use of drug classes, reinforcing the viewpoint that the incremental benefits associated with treatment were not accidental. However, this downward trend was not observed in terms of all-cause mortality. The 2021 European Society of Cardiology HF guidelines proposed the hypothesis that the lack of disease-modifying effects of drugs among patients with HFpEF was probably caused by the interference of concomitant drugs.^3^ For practical reasons, a trial confirming this hypothesis was difficult to implement, but the results of the sensitivity analysis to some degree explain this hypothesis (namely, the concomitant drug probably interfered with the disease-modifying effects of drugs in terms of HF hospitalization rather than all-cause death).

Our results showed the incremental benefits associated with drug therapies for patients with HF and an LVEF of 40% or more, which did not mean that the benefits exist for all patients with HFmrEF or HFpEF. Although patients with HFmrEF have outcomes more akin to patients with HFpEF than patients with HFrEF, the response to medical therapies among patients with HFmrEF appears to be more similar to that among patients with HFrEF.^3,4^ Thus, for patients with HF, the classification of LVEF based on patient prognosis may be inconsistent with that based on treatment response, and the patients with a relatively lower LVEF who were included in our study may have received more benefits associated with drug therapies. In 2 large-scale RCTs of HFpEF and HFmrEF, we found that the benefits associated with ARNIs and MRAs emerged primarily for patients with a baseline LVEF less than or equal to the median (57% for ARNIs; 56% for MRAs), but these benefits were weakened or even disappeared for patients with a baseline LVEF greater than the median.^17,18^ A similar situation was found for SGLT2 inhibitors.^7^ Therefore, the LVEF cutoff to classify whether a drug is effective or not for patients with HFpEF or HFmrEF may be different from the LVEF cutoff for dividing HF into HFmrEF and HFpEF, and the use of specific drugs should refer to the subgroup analysis of the LVEF in their major trials. The clinical syndrome of HFpEF is heterogeneous, which probably led to the neutral results in many clinical trials. Relative to HFrEF, the etiologic factors associated with HFpEF are more diverse and carry their own distinct pathophysiology and natural history. For the same drug treatment, people with different etiologic factors may have different treatment responses. Phenotypic classification of HFpEF is needed owing to the substantial heterogeneity, whereas there is currently no effective or uniform classification method.^6^ More studies exploring the phenotypic classification of HFpEF are still required to identify the patients who do not respond to certain drugs.

According to the baseline characteristics of the included trials, patients with HFpEF or HFmrEF, most of whom had taken ASIs and/or β-blockers before enrollment, tended to be older and to have obesity and many comorbid conditions (eg, hypertension, atrial fibrillation, and diabetes). Our primary analysis demonstrated that ACE inhibitors and ARBs were not significantly associated with a reduction in the risk of HF hospitalization for patients with HFpEF or HFmrEF. Nevertheless, eTable 2 in the Supplement showed that in 2 large included trials on ACE inhibitors and ARBs, most of the enrolled patients had taken β-blockers at baseline; thus, β-blockers were likely to interfere with the effectiveness of ACE inhibitors and ARBs. In the sensitivity analysis, after weakening the interference of concomitant β-blockers, ACE inhibitors and ARBs were associated with a significant decrease in the risk of HF hospitalization for patients with HFpEF or HFmrEF, suggesting that β-blockers were probably an interferent. We found that ASIs plus β-blockers plus SGLT2 inhibitors vs ASIs were associated with greater effectiveness than ASIs plus β-blockers plus SGLT2 inhibitors vs ASIs plus β-blockers in terms of HF hospitalization, suggesting that β-blockers may be a class of beneficial interferent and that the addition of β-blockers probably led to an incremental benefit for patients with HFpEF or HFmrEF.

Because of the weak disease-modifying effectiveness of pharmacologic therapy for patients with HF and an LVEF of 40% or more, high expectations were placed on the effectiveness of surgical treatment previously. Studies demonstrated that, for patients with HF and an LVEF of 40% or more, atrial septostomy was associated with improved quality of life and cardiac function.^19,20^ Nevertheless, a recent clinical trial suggested that atrial septostomy did not significantly improve the composite outcome or individual outcome of cardiovascular death and HF events.^21^ In the absence of disease-modifying effects of surgical treatment, proper use of disease-modifying drugs is important for the management of HFpEF and HFmrEF. In clinical practice, most patients have taken ACE inhibitors or ARBs and β-blockers to treat comorbid conditions. For these patients with HF and an LVEF of 40% or more, ARNIs may be considered to replace ACE inhibitors or ARBs if tolerated, and SGLT2 inhibitors and MRAs may be recommended to be added when indicated.

### Limitations

This study has some limitations. Owing to the internal selection bias of RCTs, their results cannot be applied to all patients with HF in clinical settings. Our analysis did not consider the doses of the included drugs, which may have had a small effect on our results. Owing to the absence of the data on subgroups of patients with HFmrEF or HFpEF in most of the included trials, we did not perform a subgroup analysis to estimate the outcomes associated with drug therapies for HFmrEF and HFpEF separately. Owing to the low number of eligible studies, other subgroup analyses were difficult to perform. Thus, we did not further explore the treatment response of patients with different phenotypes for interventions and potential heterogeneity. Because the proportion of patients taking concomitant drugs was less than 100% and may have changed slightly during the follow-up period in our sensitivity analysis, the estimated point was relatively inaccurate and served merely as a reference. The regimen containing ARNIs, MRAs, and SGLT2 inhibitors was probably the most effective therapy, but no trial on this treatment regimen was included. Future studies of SGLT2 inhibitors that include more patients with HFpEF or HFmrEF using MRAs and ARNIs are needed to explore this treatment combination.

## Conclusions

This network meta-analysis found that, for patients with HFpEF or HFmrEF, no included drug classes were significantly associated with a reduced risk of death, but SGLT2 inhibitors, ARNIs, and MRAs were associated with a significant decrease in the risk of HF admission; SGLT2 inhibitors were the optimal drug class. Our results are consistent with the latest guideline recommendations. If indicated, SGLT2 inhibitors may be preferentially recommended for patients with HFpEF or HFmrEF. The increasing use of combinations of drug therapies may be associated with accumulative benefits in terms of HF hospitalization rather than all-cause death for patients with HF and an LVEF of 40% or more. More studies that explore the phenotypic classification and the LVEF cutoff for the efficacy of drug therapies for patients with HFpEF or HFmrEF are needed.

## Notes

Supplement. **eAppendix.** Search Strategies **eFigure 1.** Flowchart of Study Selection Process **eFigure 2.** Bias Assessment of Eligible Studies **eFigure 3.** Primary Network Meta-analysis Results for All-Cause Death (A), Cardiovascular Death (B) and Hospitalization for Heart Failure (C) **eFigure 4.** Rankogram Plot for All-Cause Mortality (A), Cardiovascular Mortality (B), and Hospitalization for Heart Failure (C) in the Primary Analysis **eFigure 5.** Sensitivity Analysis Results for All-Cause Death (A) and Hospitalization for Heart Failure (B) **eFigure 6.** Rankogram Plot for All-Cause Mortality (A) and Hospitalization for Heart Failure (B) in Sensitivity Analysis **eTable 1.** Study Characteristics of Included RCTs **eTable 2.** Baseline Characteristics of Included Intervention and Concomitant Drug Classes Reported **eTable 3.** Additional Sensitivity Analysis
eReferences
