# Supplementary material for: Optimal Pharmacologic Treatment of Heart Failure With Preserved and Mildly Reduced Ejection Fraction: A Meta-analysis
Source: JAMA Netw Open. 2022 Sep 20;5(9):e2231963. doi: 10.1001/jamanetworkopen.2022.31963 (PMC9490501; doi:10.1001/jamanetworkopen.2022.31963)
Supplement: Supplement. — eAppendix. Search Strategies eFigure 1. Flowchart of Study Selection Process eFigure 2. Bias Assessment of Eligible Studies eFigure 3. Primary Network Meta-analysis Results for All-Cause Death (A), Cardiovascular Death (B) and Hospitalization for Heart Failure (C) eFigure 4. Rankogram Plot for All-Cause Mortality (A), Cardiovascular Mortality (B), and Hospitalization for Heart Failure (C) in the Primary Analysis eFigure 5. Sensitivity Analysis Results for All-Cause Death (A) and Hospitalization for Heart Failure (B) eFigure 6. Rankogram Plot for All-Cause Mortality (A) and Hospitalization for Heart Failure (B) in Sensitivity Analysis eTable 1. Study Characteristics of Included RCTs eTable 2. Baseline Characteristics of Included Intervention and Concomitant Drug Classes Reported eTable 3. Additional Sensitivity Analysis eReferences [file jamanetwopen-e2231963-s001.pdf]

Supplementary Online Content

Xiang B, Zhang R, Wu X, Zhou X. Optimal pharmacologic treatment of heart failure with preserved and mildly reduced ejection fraction: a meta-analysis. *JAMA Netw Open.* 2022;5(9):e2231963. doi:10.1001/jamanetworkopen.2022.31963

eAppendix. Search Strategies

eFigure 1. Flowchart of Study Selection Process

eFigure 2. Bias Assessment of Eligible Studies

eFigure 3. Primary Network Meta-analysis Results for All-Cause Death (A), Cardiovascular Death (B) and Hospitalization for Heart Failure (C)

eFigure 4. Rankogram Plot for All-Cause Mortality (A), Cardiovascular Mortality (B), and Hospitalization for Heart Failure (C) in the Primary Analysis

eFigure 5. Sensitivity Analysis Results for All-Cause Death (A) and Hospitalization for Heart Failure (B)

eFigure 6. Rankogram Plot for All-Cause Mortality (A) and Hospitalization for Heart Failure (B) in Sensitivity Analysis

eTable 1. Study Characteristics of Included RCTs

eTable 2. Baseline Characteristics of Included Intervention and Concomitant Drug Classes Reported

eTable 3. Additional Sensitivity Analysis

eReferences

This supplementary material has been provided by the authors to give readers additional information about their work.

© 2022 Xiang B et al. *JAMA Network Open.*

eAppendix. Search Strategies

Database	PubMed
Search time	October 9th 2021
Strategy	1. heart failure[Title/Abstract] OR cardiac failure[Title/Abstract] OR cardiac insufficiency[Title/Abstract] OR cardiomyopathy[Title/Abstract]<br>2. "Heart Failure"[Mesh]<br>3. #1 or #2<br>4. LCZ696[Title/Abstract] OR LCZ 696[Title/Abstract] OR LCZ-696[Title/Abstract] OR valsartan[Title/Abstract] OR sacubitril[Title/Abstract] OR angiotensin receptor-neprilysin inhibitor[Title/Abstract]<br>5. "Angiotensin-Converting Enzyme Inhibitors"[Mesh]<br>6. angiotensin converting enzyme inhibitor[Title/Abstract] OR ACEI[Title/Abstract] OR benazepril[Title/Abstract] OR captopril[Title/Abstract] OR enalapril[Title/Abstract] OR fosinopril[Title/Abstract] OR imidapril[Title/Abstract] OR lisinopril[Title/Abstract] OR moexipril[Title/Abstract] OR perindopril[Title/Abstract] OR quinapril[Title/Abstract] OR ramipril[Title/Abstract] OR trandolapril[Title/Abstract] OR zofenopril[Title/Abstract] OR alacepril[Title/Abstract] OR cilazapril[Title/Abstract] OR spirapril[Title/Abstract] OR delapril[Title/Abstract]<br>7. "Adrenergic beta-Antagonists"[Mesh]<br>8. beta blocker[Title/Abstract] OR BB[Title/Abstract] OR acebutolol[Title/Abstract] OR atenolol[Title/Abstract] OR betaxolol[Title/Abstract] OR bisoprolol[Title/Abstract] OR carvedilol[Title/Abstract] OR labetalol[Title/Abstract] OR metoprolol[Title/Abstract] OR nadolol[Title/Abstract] OR nebivolol[Title/Abstract] OR penbutolol[Title/Abstract] OR pindolol[Title/Abstract] OR propranolol[Title/Abstract] OR sotalol[Title/Abstract] OR timolol[Title/Abstract]<br>9. "Mineralocorticoid Receptor Antagonists"[Mesh]<br>10. aldosterone antagonist[Title/Abstract] OR mineralocorticoid-receptor antagonist[Title/Abstract] OR MRA[Title/Abstract] OR eplerenone[Title/Abstract] OR spironolactone[Title/Abstract]<br>11. "Angiotensin Receptor Antagonists"[Mesh]<br>12. angiotensin receptor blocker[Title/Abstract] OR angiotensin receptor antagonist[Title/Abstract] OR ARB[Title/Abstract] OR azilsartan[Title/Abstract] OR candesartan[Title/Abstract] OR eprosartan[Title/Abstract] OR irbesartan[Title/Abstract] OR losartan[Title/Abstract] OR olmesartan[Title/Abstract] OR telmisartan[Title/Abstract] OR valsartan[Title/Abstract]<br>13. "Sodium-Glucose Transporter 2 Inhibitors"[Mesh]<br>14. sodium-glucose cotransporter 2 inhibitor[Title/Abstract] OR sodium-glucose transporter 2 inhibitor[Title/Abstract] OR empagliflozin[Title/Abstract] OR dapagliflozin[Title/Abstract] OR canagliflozin[Title/Abstract] OR ertugliflozin[Title/Abstract] OR sotagliflozin[Title/Abstract] OR remogliflozin[Title/Abstract] OR ipragliflozin[Title/Abstract] OR tofogliflozin[Title/Abstract]<br><br>15. #4 or #5 or #6 or #7 or #8 or #9 or #10 or #11 or #12 or #13 or #14<br>16. #3 and #15

© 2022 Xiang B et al. *JAMA Network Open.*

	17. #16 AND ((clinicaltrial[Filter]) AND (humans[Filter]))
Database	EMBASE
Search time	October 9th 2021
Strategy	1. 'heart failure'/exp<br>2. 'heart failure':ti,ab,kw OR 'cardiac failure':ti,ab,kw OR 'cardiac insufficiency':ti,ab,kw OR cardiomyopathy:ti,ab,kw<br>3. #1 OR #2<br>4. lcz696:ti,ab,kw OR 'lcz 696':ti,ab,kw OR valsartan:ti,ab,kw OR sacubitril:ti,ab,kw OR 'angiotensin receptor-neprilysin inhibitor':ti,ab,kw<br>5. 'dipeptidyl carboxypeptidase inhibitor'/exp<br>6. 'angiotensin converting enzyme inhibitor':ti,ab,kw OR acei:ti,ab,kw OR benazepril:ti,ab,kw OR captopril:ti,ab,kw OR enalapril:ti,ab,kw OR fosinopril:ti,ab,kw OR imidapril:ti,ab,kw OR lisinopril:ti,ab,kw OR moexipril:ti,ab,kw OR perindopril:ti,ab,kw OR quinapril:ti,ab,kw OR ramipril:ti,ab,kw OR trandolapril:ti,ab,kw OR zofenopril:ti,ab,kw OR alacepril:ti,ab,kw OR cilazapril:ti,ab,kw OR spirapril:ti,ab,kw OR delapril:ti,ab,kw<br>7. 'adrenergic receptor blocking agent'/exp<br>8. 'beta blocker':ti,ab,kw OR bb:ti,ab,kw OR acebutolol:ti,ab,kw OR atenolol:ti,ab,kw OR betaxolol:ti,ab,kw OR bisoprolol:ti,ab,kw OR carvedilol:ti,ab,kw OR labetalol:ti,ab,kw OR metoprolol:ti,ab,kw OR nadolol:ti,ab,kw OR nebivolol:ti,ab,kw OR penbutolol:ti,ab,kw OR pindolol:ti,ab,kw OR propranolol:ti,ab,kw OR sotalol:ti,ab,kw OR timolol:ti,ab,kw<br>9. 'mineralocorticoid antagonist'/exp<br>10. 'aldosterone antagonist':ti,ab,kw OR 'mineralocorticoid-receptor antagonist':ti,ab,kw OR mra:ti,ab,kw OR eplerenone:ti,ab,kw OR spironolactone:ti,ab,kw<br>11. 'angiotensin receptor antagonist'/exp<br>12. 'angiotensin receptor blocker':ti,ab,kw OR 'angiotensin receptor antagonist':ti,ab,kw OR arb:ti,ab,kw OR azilsartan:ti,ab,kw OR candesartan:ti,ab,kw OR eprosartan:ti,ab,kw OR irbesartan:ti,ab,kw OR losartan:ti,ab,kw OR olmesartan:ti,ab,kw OR telmisartan:ti,ab,kw OR valsartan:ti,ab,kw<br>13. 'sodium glucose cotransporter 2 inhibitor'/exp<br>OR ertugliflozin:ti,ab,kw OR sotagliflozin:ti,ab,kw OR remogliflozin:ti,ab,kw OR ipragliflozin:ti,ab,kw OR tofogliflozin:ti,ab,kw<br>14. 'sodium-glucose cotransporter 2 inhibitor':ti,ab,kw OR 'sodium-glucose transporter 2 inhibitor':ti,ab,kw OR empagliflozin:ti,ab,kw OR dapagliflozin:ti,ab,kw OR canagliflozin:ti,ab,kw<br>15. #4 OR #5 OR #6 OR #7 OR #8 OR #9 OR #10 OR #11 OR #12 OR #13 OR #14<br>16. #3 AND #15<br>17. #16 AND ([adult]/lim OR [aged]/lim) AND 'clinical trial'/de
Database	CENTRAL
Search time	October 9th 2021
Strategy	1. MeSH descriptor: [Heart Failure] explode all trees<br>2. (heart failure or cardiac failure or cardiac insufficiency or cardiomyopathy):ti,ab,kw<br>3. #1 or #2

© 2022 Xiang B et al. *JAMA Network Open.*

	4. LCZ696 or LCZ 696 or LCZ-696 or valsartan or sacubitril or angiotensin receptor-neprilysin inhibitor):ti,ab,kw 2500<br>5. MeSH descriptor: [Angiotensin-Converting Enzyme Inhibitors] explode all trees<br>6. (angiotensin converting enzyme inhibitor or ACEI or ACEI or benazepril or captopril or enalapril or fosinopril or imidapril or lisinopril or moexipril or perindopril or quinapril or ramipril or trandolapril or zofenopril or alacepril or cilazapril or spirapril or delapril):ti,ab,kw<br>7.MeSH descriptor: [Adrenergic beta-Antagonists] explode all trees<br>8. (beta blocker or BB or acebutolol or atenolol or betaxolol or bisoprolol or carvedilol or labetalol or metoprolol or nadolol or nebivolol or penbutolol or pindolol or propranolol or sotalol or timolol):ti,ab,kw<br>9. MeSH descriptor: [Mineralocorticoid Receptor Antagonists] explode all trees<br>10. (aldosterone antagonist or mineralocorticoid-receptor antagonist or MRA or eplerenone or spironolactone):ti,ab,kw<br>11. MeSH descriptor: [Angiotensin Receptor Antagonists] explode all trees<br>12. (angiotensin receptor blocker or angiotensin receptor antagonist or ARB or azilsartan or candesartan or eprosartan or irbesartan or losartan or olmesartan or telmisartan or valsartan):ti,ab,kw<br>13.MeSH descriptor: [Sodium-Glucose Transporter 2 Inhibitors] explode all trees<br>14. (sodium-glucose cotransporter 2 inhibitor or sodium-glucose transporter 2 inhibitor or empagliflozin or dapagliflozin or canagliflozin or ertugliflozin or sotagliflozin or remogliflozin or ipragliflozin or tofogliflozin):ti,ab,kw<br>15. #4 or #5 or #6 or #7 or #8 or #9 or #10 or #11 or #12 or #13 or #14<br>16. #3 and #15<br>17. human not animal<br>18. #16 and #17, in Trials

© 2022 Xiang B et al. *JAMA Network Open.*

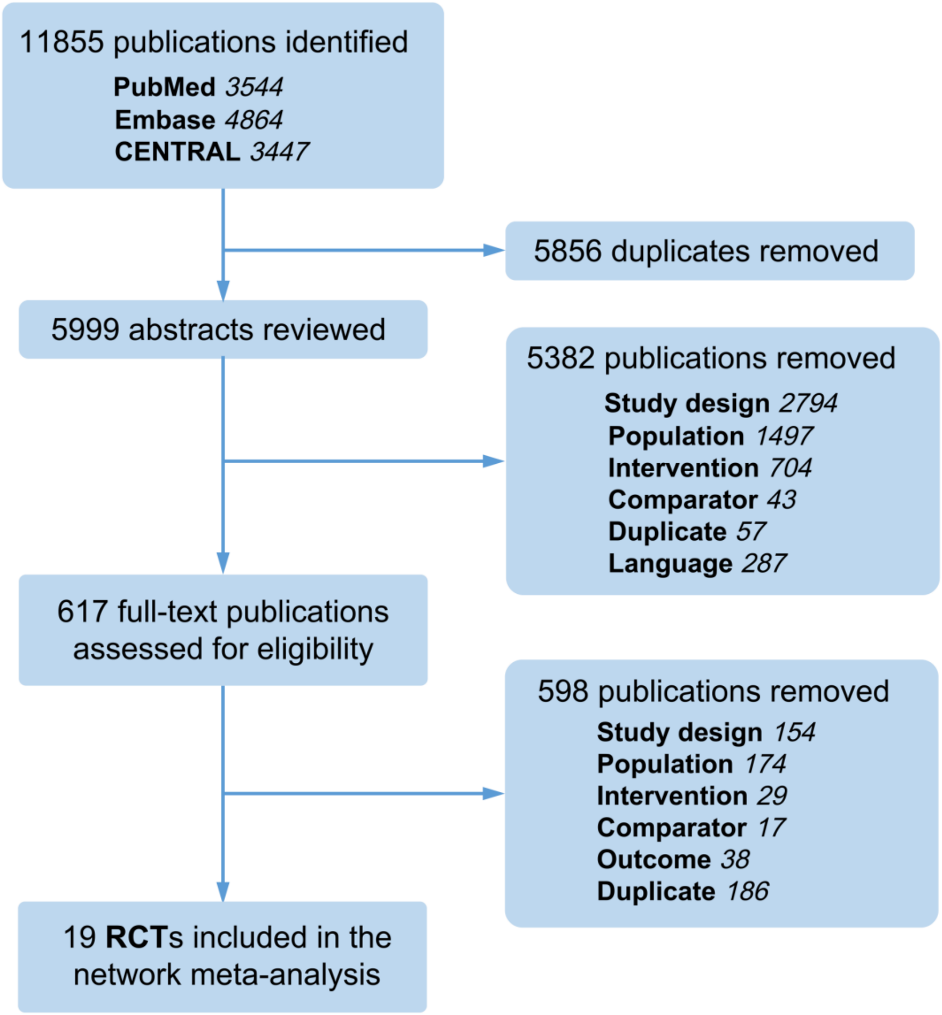

eFigure 1. Flowchart of Study Selection Process

© 2022 Xiang B et al. *JAMA Network Open.*

Trial/Author year	Random sequence generation	Allocation concealment	Blinding of participants and personnel	Blinding of outcome assessment	Incomplete outcome data	Selective reporting	Other bias
Aldo-DHF 2013	Low	Low	Low	Low	Low	Low	Low
CHARM-Preserved 2003	Unclear	Low	Low	Low	Low	Low	Low
ELANDD 2012	Low	Unclear	Low	Unclear	Low	Low	Low
EMPEROR-Preserved 2021	Low	Low	Low	Low	Low	Low	Low
J-DHF 2013	Unclear	Unclear	High	Low	Low	Low	Low
Kasama 2004	Unclear	Unclear	Low	Low	High	Low	Low
Kitzman 2010	Unclear	Unclear	Low	Low	Unclear	Unclear	Low
Parthasarathy 2009	Low	Low	Low	Low	Low	Low	Low
McDiarmid 2019	Low	Low	High	High	High	Low	Low
PARAGON-HF 2019	Low	Low	Low	Low	Low	Low	Low
PARAMOUNT 2012	Low	Low	Low	Low	Low	Low	Low
PEP-CHF 2006	Low	Low	Low	Low	Low	Low	Low
SENIORS 2009	Low	Low	Low	Unclear	Low	Low	Low
STRUCTURE 2016	Unclear	Low	Low	Low	Low	Unclear	Low
SWEDIC 2004	Unclear	Unclear	Low	Unclear	Unclear	Unclear	Low
Takeda 2004	Unclear	Unclear	Unclear	Unclear	Unclear	Unclear	Unclear
TOPCAT 2014	Low	Low	Low	Low	Low	Low	Low
Yip 2007	Low	Low	High	Low	Unclear	Low	Low
Zi 2003	Unclear	Unclear	Low	Unclear	Low	Unclear	Low

eFigure 2. Bias Assessment of Eligible Studies

© 2022 Xiang B et al. *JAMA Network Open.*

A

SGLT2i						
1.02<br>(0.77-1.32)<br>0.10	ARNI					
1.10<br>(0.88-1.36)<br>0.61	1.10<br>(0.81-1.45)<br>0.46	MRA				
1.09<br>(0.74-1.54)<br>0.33	1.08<br>(0.70-1.59)<br>0.28	0.99<br>(0.66-1.42)<br>0.02	BB			
0.98<br>(0.78-1.23)<br>0.11	0.97<br>(0.83-1.12)<br>0.30	0.90<br>(0.70-1.14)<br>0.60	0.93<br>(0.63-1.35)<br>0.26	ARB		
0.98<br>(0.65-1.42)<br>0.07	0.98<br>(0.62-1.47)<br>0.07	0.90<br>(0.59-1.32)<br>0.39	0.93<br>(0.55-1.49)<br>0.20	1.01<br>(0.66-1.48)<br>0.03	ACEI	
1.00<br>(0.87-1.15)<br>0.03	1.00<br>(0.79-1.25)<br>0.01	0.92<br>(0.77-1.08)<br>0.68	0.95<br>(0.67-1.32)<br>0.20	1.03<br>(0.86-1.23)<br>0.23	1.06<br>(0.73-1.50)<br>0.24	PLBO
I^2=0%; P>0.05						

B

SGLT2i						
1.02<br>(0.71-1.42)<br>0.09	ARNI					
1.02<br>(0.76-1.34)<br>0.11	1.02<br>(0.70-1.44)<br>0.09	MRA				
1.09<br>(0.65-1.72)<br>0.25	1.09<br>(0.62-1.80)<br>0.23	1.08<br>(0.63-1.72)<br>0.22	BB			
0.97<br>(0.72-1.27)<br>0.18	0.95<br>(0.78-1.15)<br>0.35	0.96<br>(0.70-1.28)<br>0.21	0.94<br>(0.55-1.49)<br>0.19	ARB		
0.99<br>(0.60-1.54)<br>0.03	0.99<br>(0.57-1.61)<br>0.02	0.98<br>(0.58-1.54)<br>0.06	0.96<br>(0.49-1.71)<br>0.10	1.04<br>(0.62-1.63)<br>0.11	ACEI	
0.91<br>(0.76-1.09)<br>0.67	0.92<br>(0.68-1.21)<br>0.43	0.91<br>(0.73-1.12)<br>0.63	0.89<br>(0.55-1.35)<br>0.39	0.96<br>(0.77-1.19)<br>0.28	0.97<br>(0.61-1.48)<br>0.08	PLBO
I^2=0%; P>0.05						

C

SGLT2i						
0.94<br>(0.71-1.23)<br>0.31	ARNI					
0.86<br>(0.67-1.09)<br>0.77	0.93<br>(0.69-1.22)<br>0.39	MRA				
0.96<br>(0.50-1.68)<br>0.11	1.03<br>(0.52-1.83)<br>0.06	1.12<br>(0.58-1.97)<br>0.27	BB			
0.85<br>(0.67-1.07)<br>0.81	0.91<br>(0.79-1.04)<br>0.84	1.00<br>(0.77-1.27)<br>0.02	0.96<br>(0.50-1.64)<br>0.11	ARB		
0.86<br>(0.60-1.21)<br>0.58	0.93<br>(0.62-1.33)<br>0.29	1.01<br>(0.69-1.43)<br>0.04	0.97<br>(0.48-1.75)<br>0.06	1.02<br>(0.71-1.44)<br>0.09	ACEI	
0.71<br>***(0.60-0.83)***<br>***1.00***	***0.76***<br>***(0.61-0.95)***<br>***0.98***	***0.83***<br>***(0.69-0.99)***<br>***0.95***	0.82<br>(0.44-1.40)<br>0.48	0.84<br>(0.71-1.00)<br>0.94	0.85<br>(0.61-1.15)<br>0.69	PLBO
I^2=0%; P>0.05						

eFigure 3. Primary Network Meta-Analysis Results for All-Cause Death (A), Cardiovascular Death (B) and Hospitalization for Heart Failure (C). The hazard ratio, 95% credible interval, and the probability (P score) that the intervention is more effective than the comparator are presented for each pairwise comparison. Results are shown in bold and italics if they are statistically significant. No significant heterogeneity (I^2) or inconsistency (*P*-value) for three outcomes was found.

© 2022 Xiang B et al. *JAMA Network Open.*

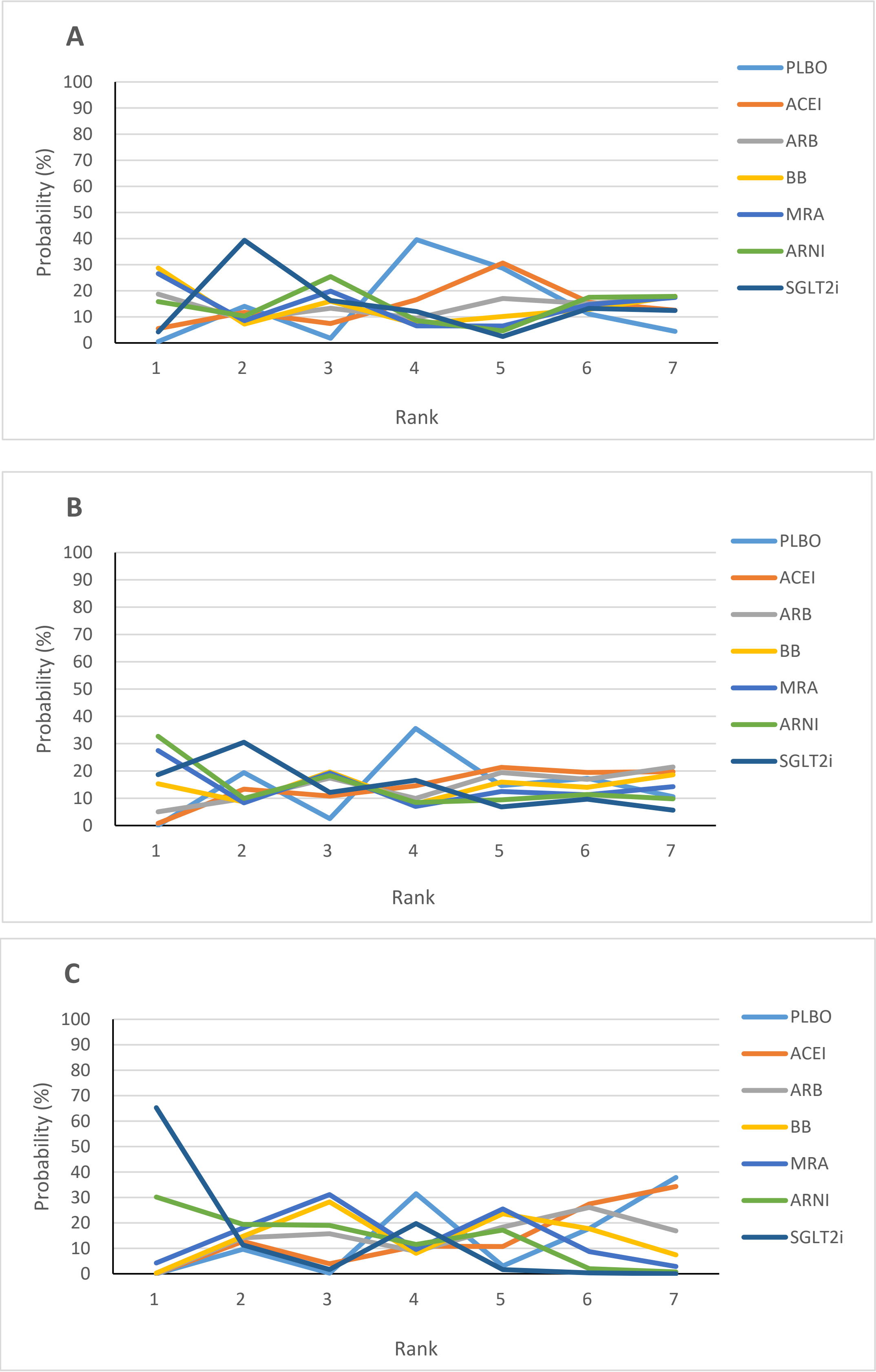

eFigure 4. Rankogram Plot for All-Cause Mortality (A), Cardiovascular Mortality (B), and Hospitalization for Heart Failure (C) in the Primary Analysis.

© 2022 Xiang B et al. *JAMA Network Open.*

A

**ASI+BB +SGLT2i**						
1.10 (0.88-1.36)<br>0.61	**ASI+BB +MRA**					
1.04 (0.84-1.27)<br>0.27	0.95 (0.75-1.18)<br>0.32	**ARNI+BB**				
1.00 (0.87-1.15)<br>0.03	0.92 (0.77-1.08)<br>0.69	0.97 (0.83-1.12)<br>0.30	**ASI+BB**			
1.05 (0.84-1.29)<br>0.31	0.96 (0.75-1.20)<br>0.28	1.01 (0.81-1.25)<br>0.07	1.04 (0.88-1.22)<br>0.38	**BB**		
0.95 (0.65-1.34)<br>0.19	0.87 (0.59-1.25)<br>0.52	0.92 (0.63-1.31)<br>0.32	0.95 (0.67-1.31)<br>0.22	0.92 (0.62-1.31)<br>0.34	**ASI**	
0.31 (0.03-1.11)<br>0.78	0.28 (0.02-1.02)<br>0.81	0.30 (0.03-1.07)<br>0.79	0.31 (0.03-1.10)<br>0.78	0.30 (0.03-1.08)<br>0.79	0.33 (0.03-1.16)<br>0.76	**PLBO**
I^2=0%; P=NA						

B

**ASI+BB +SGLT2i**						
0.86 (0.67-1.09)<br>0.77	**ASI+BB +MRA**					
0.79 (0.64-0.97)<br>***0.97***	0.92 (0.73-1.15)<br>0.50	**ARNI+BB**				
0.71 (0.60-0.83)<br>***1.00***	***0.83 (0.69-0.99)***<br>***0.95***	0.91 (0.79-1.04)<br>0.84	**ASI+BB**			
0.60 (0.48-0.75)<br>***1.00***	***0.71 (0.55-0.89)***<br>***1.00***	***0.77 (0.62-0.94)***<br>***0.99***	***0.85 (0.72-0.99)***<br>***0.96***	**BB**		
0.57 (0.30-0.98)<br>***0.93***	0.67 (0.35-1.15)<br>0.81	0.73 (0.38-1.24)<br>0.71	0.80 (0.43-1.34)<br>0.54	0.95 (0.50-1.64)<br>0.11	**ASI**	
0.46 (0.15-1.12)<br>0.87	0.53 (0.17-1.31)<br>0.77	0.58 (0.19-1.41)<br>0.71	0.64 (0.21-1.55)<br>0.61	0.76 (0.25-1.85)<br>0.39	0.80 (0.32-1.70)<br>0.39	**PLBO**
I^2=0%; P=NA						

eFigure 5. Sensitivity Analysis Results for All-Cause Death (A) and Hospitalization for Heart Failure (B). The hazard ratio, 95% credible interval, and the probability (P score) that the intervention is more effective than the comparator are presented for each pairwise comparison. Results are shown in bold and italics if they are statistically significant. No significant heterogeneity (I^2) for two outcomes was found, and the inconsistency (*P*-value) was not available (NA).

© 2022 Xiang B et al. *JAMA Network Open.*

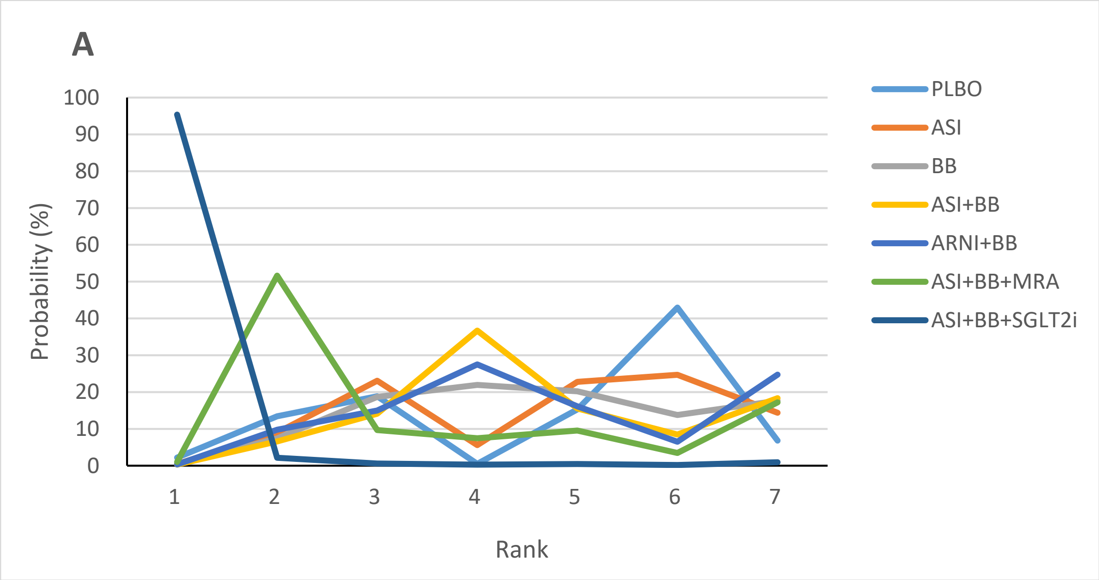

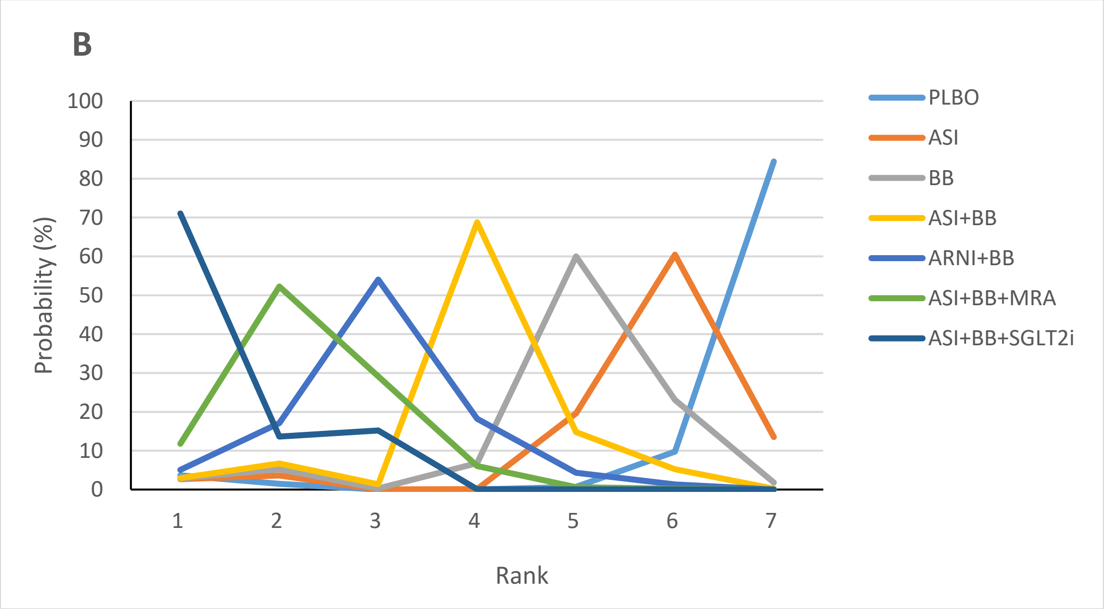

eFigure 6. Rankogram Plot for All-Cause Mortality (A) and Hospitalization for Heart Failure (B) in Sensitivity Analysis.

© 2022 Xiang B et al. *JAMA Network Open.*

eTable 1. Study Characteristics of Included RCTs

Trial/Author year	Study design	LVEF inclusion criteria	Median follow-up (moth)	Treatment	Daily dose	Number	Mean					Female (%)	NYHA (%)				Ischemic HF (%)	HT (%)	AF (%)	DM (%)
							Age (year)	BMI (kg/m^2)	HR (bpm)	LVEF (%)	eGFR (ml/min/ 1.73 m^2)		I	II	III	IV				
**PARAGON-HF 2019[1]**	DB, MC	≥45%	35.0	Sacubitril-valsartan	400 mg	2407	72.7	30.2	70.6	57.6	63.0	51.6	3.0	77.5	19.0	0.3	37.4	95.7	32.2	43.5
				Valsartan	320 mg	2389	72.8	30.3	70.3	57.5	62.0	51.8	2.7	77.0	19.8	0.5	34.5	95.4	32.5	42.5
**PARAMOUNT 2012[2]**	DB, MC, PC	≥45%	9.0	LCZ696	400 mg	149	70.9	30.1	69.0	58.0	67.0	57.0	1.0	81.0	19.0	0.0	NA	95.0	27.0	41.0
				Valsartan	320 mg	152	71.2	29.8	70.0	58.0	64.0	56.0	1.0	78.0	21.0	0.0	NA	92.0	30.0	35.0
**EMPEROR-Preserved 2021[3]**	DB, MC, PC	> 40%	26.2	Empagliflozin	10 mg	2997	71.8	29.8	70.4	54.3	60.6	44.6	0.1	81.1	18.4	0.3	36.0	90.8	51.5	48.9
				Placebo	NA	2991	71.9	29.9	70.3	54.3	60.6	44.7	0.1	81.9	17.8	0.3	34.7	90.4	50.6	49.2
**CHARM-Preserved 2003[4]**	DB, MC, PC	> 40%	36.3	Candesartan	32 mg	1514	67.2	29.3	71.2	54.0	NA	39.2	0.0	61.5	36.7	1.8	56.4	65.0	29.0	28.7
				Placebo	NA	1509	67.1	29.0	71.4	54.1	NA	41.0	0.0	60.0	38.7	1.3	56.5	63.6	29.3	28.0
**Kasama 2004[5]**	DB, PC	> 40%	6.0	Candesartan	8-12 mg	25	66.0	NA	NA	54.0	NA	32.0	0.0	64.0	36.0	0.0	0.0	64.0	NA	NA
				Placebo	NA	25	67.0	NA	NA	55.0	NA	36.0	0.0	68.0	32.0	0.0	0.0	60.0	NA	NA
**Parthasarathy 2009[6]**	DB, MC, PC	≥40%	3.5	Valsartan	320 mg	68	61.0	31.0	NA	70.5	NA	50.0	NA	NA	NA	NA	NA	91.2	16.2	22.1
				Placebo	NA	82	63.1	29.3	NA	71.5	NA	50.0	NA	NA	NA	NA	NA	89.0	9.8	14.6
**TOPCAT 2014[7]**	DB, MC, PC	≥45%	39.6	Spironolactone	15-45 mg	1722	68.7	31.0	68.0	56.0	65.3	51.6	3.3	63.3	33.0	0.4	NA	91.0	35.5	32.8
				Placebo	NA	1723	68.7	31.0	68.0	56.0	65.5	51.5	3.1	64.1	32.1	0.5	NA	91.9	35.1	32.2
**Aldo-DHF 2013[8]**	DB, MC, PC	≥50%	11.6	Spironolactone	25 mg	213	67.0	28.9	66.0	67.0	79.0	52.0	0.0	85.0	15.0	0.0	NA	92.0	6.0	17.0
				Placebo	NA	209	67.0	28.9	64.0	68.0	78.0	53.0	0.0	88.0	12.0	0.0	NA	91.0	4.0	16.0
**McDiarmid 2019[9]**	DB, MC, PC	> 50%	6.0	Spironolactone	25 mg	19	76.4	29.8	77.0	53.5	NA	47.4	0.0	73.7	26.3	0.0	NA	79.0	89.0	NA
				Placebo	NA	21	74.0	29.1	74.5	54.8	NA	52.4	0.0	81.0	19.0	0.0	NA	62.0	71.0	NA
**STRUCTURE 2016[10]**	DB, SC, PC	> 50%	6.0	Spironolactone	25 mg	64	66.3	30.7	72.0	72.6	64.3	88.0	0.0	78.0	22.0	0.0	NA	92.0	NA	39.0
				Placebo	NA	67	67.6	29.7	73.0	71.4	63.5	81.0	0.0	79.0	21.0	0.0	NA	91.0	NA	40.0

© 2022 Xiang B et al. *JAMA Network Open.*

**PEP-CHF 2006[11]**	DB, MC, PC	NA	26.2	Perindopril	4 mg	424	75.0	27.5	74.0	65.0	NA	54.0	NA	NA	NA	NA	NA	79.0	19.0	21.0
				Placebo	NA	426	75.0	27.6	73.0	64.0	NA	57.0	NA	NA	NA	NA	NA	79.0	22.0	20.0
**Kitzman 2010[12]**	DB, PC	≥50%	12.0	Enalapril	20 mg	35	69.0	30.0	NA	65.0	NA	80.0	0.0	83.0	17.0	0.0	NA	71.0	NA	9.0
				Placebo	NA	36	70.0	30.0	NA	65.0	NA	89.0	0.0	75.0	25.0	0.0	NA	75.0	NA	17.0
**Yip 2007[13]**	DB, MC, PC	> 45%	12.0	Ramipril+Diuretic	R:10 mg; D: NA	45	74.0	26.8	79.0	65.0	NA	60.0	0.0	66.7	33.3	0.0	16.0	73.0	16.0	22.0
				Irbesartan+Diuretic	I:75 mg; D:NA	56	75.0	27.2	77.0	66.0	NA	66.0	0.0	67.9	30.4	0.0	7.0	71.0	21.0	18.0
				Diuretic	NA	50	73.0	26.8	76.0	69.0	NA	58.0	0.0	72.0	28.0	0.0	12.0	76.0	10.0	20.0
**Zi 2003[14]**	DB, MC, PC	≥40%	6.0	Quinapril	5-40 mg	36	77.0	28.5	NA	59.3	NA	61.1	5.5	77.8	16.7	0.0	NA	27.8	38.9	11.1
				Placebo	NA	38	78.0	27.0	NA	58.0	NA	68.4	0.0	73.7	26.3	0.0	NA	31.6	31.6	18.4
**ELANDD 2012[15]**	DB, MC, PC	> 45%	6.0	Nebivolol	5-10 mg	57	66.5	30.3	NA	61.9	NA	65.0	0.0	77.0	23.0	0.0	17.0	86.0	NA	21.0
				Placebo	NA	59	65.3	30.2	NA	63.2	NA	64.0	0.0	78.0	22.0	0.0	20.0	86.4	NA	20.0
**J-DHF 2013[16]**	DB, MC, PC	> 40%	38.4	Carvedilol	2.5-20 mg	120	73.0	24.2	72.0	62.0	58.0	42.5	18.3	69.2	10.8	2.0	17.5	80.0	50.8	27.5
				Placebo	NA	125	71.0	24.1	74.0	63.0	58.3	41.6	18.4	75.2	4.8	1.6	12.0	80.8	45.6	33.6
**SWEDIC 2004[17]**	DB, MC, PC	> 45%	6.0	Carvedilol	50-100 mg	47	67.0	NA	74.0	NA	NA	40.4	40.0	53.0	7.0	0.0	NA	70.2	NA	12.8
				Placebo	NA	50	66.0	NA	73.0	NA	NA	46.0	26.0	53.0	21.0	0.0	NA	62.0	NA	16.0
**Takeda 2004[18]**	SC	≥45%	12.0	Carvedilol	≥5 mg	19	69.1	NA	70.6	55.8	NA	32.0	0.0	63.2	36.8	0.0	NA	63.0	21.0	NA
				No treatment	NA	21	73.1	NA	68.9	57.5	NA	62.0	0.0	71.4	28.6	0.0	NA	57.0	38.0	NA
**SENIORS 2009[19]**	DB, MC, PC	≥40%	21.0	Nebivolol	10 mg	320	76.0	NA	78.1	49.3	68.7	50.8	4.0	62.1	32.4	1.6	NA	76.6	35.0	22.9
				Placebo	NA	323	76.2	NA	78.4	49.1	66.7	48.9	3.0	62.9	32.0	2.2	NA	78.8	37.1	25.8

Abbreviations: AF, atrial fibrillation; BMI, body mass index; DB, double blind; DM, diabetes mellitus; eGFR, estimated glomerular filtration rate; HF, heart failure; HR, heart rate; HT, hypertension; LVEF, left ventricular ejection fraction; MC, multi-center; NA, not available; NYHA, New York Heart Association; PC, placebo controlled; SC, single centre.

© 2022 Xiang B et al. *JAMA Network Open.*

eTable 2. Baseline Characteristics of Included Intervention and Concomitant Drug Classes Reported

Trial/Author year	Intervention class	Treatment	ACEI (%)	ARB (%)	BB (%)	MRA (%)
**PARAGON-HF 2019**	ARNI+BB	Sacubitril-valsartan	86.2*	86.2*	79.9	24.6
	ASI+BB	Valsartan	86.4*	86.4*	79.5	27.1
**PARAMOUNT 2012**	ARNI+BB	LCZ696	56.0	38.0	79.0	19.0
	ASI+BB	Valsartan	53.0	41.0	80.0	23.0
**EMPEROR-Preserved 2021**	SGLT2i+ASI+BB	Empagliflozin	78.8*	78.8*	86.7	37.3
	ASI+BB	Placebo	78.1*	78.1*	85.9	37.6
**CHARM-Preserved 2003**	ASI+BB	Candesartan	19.6	NA	55.9	11.3
	BB	Placebo	18.6	NA	55.5	12.0
**Kasama 2004**	ASI	Candesartan	92.0	NA	12.0	16.0
	PLBO	Placebo	96.0	NA	12.0	20.0
**Parthasarathy 2009**	ASI	Valsartan	41.2	NA	33.8	NA
	PLBO	Placebo	37.9	NA	34.1	NA
**TOPCAT 2014**	ASI+BB+MRA	Spironolactone	84.3*	84.3*	78.2	NA
	ASI+BB	Placebo	84.2*	84.2*	77.3	NA
**Aldo-DHF 2013**	ASI+BB+MRA	Spironolactone	78.0*	78.0*	69.0	NA
	ASI+BB	Placebo	76.0*	76.0*	75.0	NA
**McDiarmid 2019**	ASI+BB+MRA	Spironolactone	58.0*	58.0*	53.0	NA
	ASI+BB	Placebo	57.0*	57.0*	67.0	NA
**STRUCTURE 2016**	ASI+BB+MRA	Spironolactone	97.0*	97.0*	78.0	NA
	ASI+BB	Placebo	95.0*	95.0*	72.0	NA
**PEP-CHF 2006**	ASI+BB	Perindopril	NA	NA	55.0	9.0
	BB	Placebo	NA	NA	54.0	11.0
**Kitzman 2010**	ASI	Enalapril	NA	NA	29.0	NA
	PLBO	Placebo	NA	NA	39.0	NA
**Yip 2007**	ASI	Ramipril+Diuretic	NA	NA	NA	NA
	ASI	Irbesartan+Diuretic	NA	NA	NA	NA
	PLBO	Diuretic	NA	NA	NA	NA
**Zi 2003**	ASI	Quinapril	NA	NA	19.4	NA
	PLBO	Placebo	NA	NA	7.9	NA
**ELANDD 2012**	ASI+BB	Nebivolol	75.0*	75.0*	NA	NA

© 2022 Xiang B et al. *JAMA Network Open.*

	ASI	Placebo	80.0*	80.0*	NA	NA
**J-DHF 2013**	ASI+BB	Carvedilol	24.2	50.8	NA	20.8
	ASI	Placebo	22.4	56.0	NA	25.6
**SWEDIC 2004**	ASI+BB	Carvedilol	>50%	NA	NA	NA
	ASI	Placebo	>50%	NA	NA	NA
**Takeda 2004**	ASI+BB	Carvedilol	79.0	NA	NA	NA
	ASI	No treatment	86.0	NA	NA	NA
**SENIORS 2009**	ASI+BB	Nebivolol	86.8	5.8	NA	20.3
	ASI	Placebo	85.0	5.4	NA	16.9

* indicates the combined reporting of the use of ACEI and ARB. Abbreviations: ACEI, angiotensin-converting enzyme inhibitor; ARB, angiotensin receptor blocker; ARNI, angiotensin receptor-neprilysin inhibitor; ASI, angiotensin system inhibitor (ACEI/ARB); BB, beta-blocker; HF, heart failure; MRA, mineralocorticoid receptor antagonist; NA, not available; PLBO, placebo; SGLT2i, sodium-glucose cotransporter 2 inhibitor.

© 2022 Xiang B et al. *JAMA Network Open.*

eTable 3. Additional Sensitivity Analysis

	Kasama 2004	Kitzman 2010	Zi 2003	Takeda 2004	No removal
SGLT2i	0.71 (0.60-0.84)	0.71 (0.60-0.83)	0.71 (0.60-0.83)	0.71 (0.60-0.83)	0.71 (0.60-0.83)
ARNI	0.77 (0.61-0.95)	0.76 (0.61-0.95)	0.77 (0.61-0.95)	0.76 (0.61-0.95)	0.76 (0.61-0.95)
MRA	0.83 (0.69-0.99)	0.83 (0.69-0.99)	0.83 (0.69-0.99)	0.83 (0.69-0.99)	0.83 (0.69-0.99)
BB	0.80 (0.44-1.34)	0.80 (0.44-1.34)	0.80 (0.44-1.34)	0.82 (0.44-1.39)	0.82 (0.44-1.40)
ARB	0.84 (0.71-1.00)	0.84 (0.71-1.00)	0.84 (0.71-1.00)	0.84 (0.70-1.00)	0.84 (0.71-1.00)
ACEI	0.85 (0.61-1.15)	0.85 (0.61-1.15)	0.87 (0.63-1.19)	0.85 (0.61-1.15)	0.85 (0.61-1.15)
PLBO	1.00	1.00	1.00	1.00	1.00

The network meta-analysis results for HF hospitalization after removing certain studies with small sample sizes (n <100) are presented. Abbreviations: ACEI, angiotensin-converting enzyme inhibitor; ARB, angiotensin receptor blocker; ARNI, angiotensin receptor-neprilysin inhibitor; BB, beta-blocker; MRA, mineralocorticoid receptor antagonist; PLBO, placebo; SGLT2i, sodium-glucose cotransporter 2 inhibitor.

© 2022 Xiang B et al. *JAMA Network Open.*

eReferences

1. Solomon SD, McMurray JJV, Anand IS, Ge J, Lam CSP. Angiotensin-Neprilysin Inhibition in Heart Failure with Preserved Ejection Fraction. *N Engl J Med.* 2019; 381:1609-1620.

2. Solomon SD, Zile M, Pieske B, Voors A, Shah A. The angiotensin receptor neprilysin inhibitor LCZ696 in heart failure with preserved ejection fraction: a phase 2 double-blind randomised controlled trial. *Lancet.* 2012;380:1387-1395.

3. Anker SD, Butler J, Filippatos G, Ferreira JP, Bocchi E. Empagliflozin in Heart Failure with a Preserved Ejection Fraction. *N Engl J Med.* 2021;385:1451-1461.

4. Yusuf S, Pfeffer MA, Swedberg K, Granger CB, Held P. Effects of candesartan in patients with chronic heart failure and preserved left-ventricular ejection fraction: the CHARM-Preserved Trial. *Lancet.* 2003;362:777-781.

5. Kasama S, Toyama T, Kumakura H, Takayama Y, Ichikawa S. Effects of candesartan on cardiac sympathetic nerve activity in patients with congestive heart failure and preserved left ventricular ejection fraction. *J Am Coll Cardiol.* 2005;45:661-667.

6. Parthasarathy HK, Pieske B, Weisskopf M, Andrews CD, Brunel P. A randomized, double-blind, placebo-controlled study to determine the effects of valsartan on exercise time in patients with symptomatic heart failure with preserved ejection fraction. *Eur J Heart Fail.* 2009;11:980-989.

7. Pitt B, Pfeffer MA, Assmann SF, Boineau R, Anand IS. Spironolactone for heart failure with preserved ejection fraction. *N Engl J Med.* 2014;370:1383-1392.

8. Edelmann F, Wachter R, Schmidt AG, Kraigher-Krainer E, Colantonio C. Effect of spironolactone on diastolic function and exercise capacity in patients with heart failure with preserved ejection fraction: the Aldo-DHF randomized controlled trial. *Jama.* 2013;309:781-791.

9. McDiarmid AK, Swoboda PP, Erhayiem B, Bounford KA, Bijsterveld P. Myocardial Effects of Aldosterone Antagonism in Heart Failure With Preserved Ejection Fraction. *J Am Heart Assoc.* 2020;9:e011521.

10. Kosmala W, Rojek A, Przewlocka-Kosmala M, Wright L, Mysiak A, Marwick TH. Effect of Aldosterone Antagonism on Exercise Tolerance in Heart Failure With Preserved Ejection Fraction. *J Am Coll Cardiol.* 2016;68:1823-1834.

11. Cleland JG, Tendera M, Adamus J, Freemantle N, Polonski L, Taylor J. The perindopril in elderly people with chronic heart failure (PEP-CHF) study. *Eur Heart J.* 2006;27:2338-2345.

12. Kitzman DW, Hundley WG, Brubaker PH, Morgan TM, Moore JB. A randomized double-blind trial of enalapril in older patients with heart failure and preserved ejection fraction: effects on exercise tolerance and arterial distensibility. *Circ Heart Fail.* 2010;3:477-485.

13. Yip GW, Wang M, Wang T, Chan S, Fung JW. The Hong Kong diastolic heart failure study: a randomised controlled trial of diuretics, irbesartan and ramipril on quality of life, exercise capacity, left ventricular global and regional function in heart failure with a normal ejection fraction. *Heart.* 2008;94:573-580.

14. Zi M, Carmichael N, Lye M. The effect of quinapril on functional status of elderly patients with diastolic heart failure. *Cardiovasc Drugs Ther.* 2003;17:133-139.

© 2022 Xiang B et al. *JAMA Network Open.*

15. Conraads VM, Metra M, Kamp O, De Keulenaer GW, Pieske B. Effects of the long-term administration of nebivolol on the clinical symptoms, exercise capacity, and left ventricular function of patients with diastolic dysfunction: results of the ELANDD study. *Eur J Heart Fail.* 2012;14:219-225.

16. Yamamoto K, Origasa H, Hori M. Effects of carvedilol on heart failure with preserved ejection fraction: the Japanese Diastolic Heart Failure Study (J-DHF). *Eur J Heart Fail.* 2013;15:110-118.

17. Bergström A, Andersson B, Edner M, Nylander E, Persson H, Dahlström U. Effect of carvedilol on diastolic function in patients with diastolic heart failure and preserved systolic function. Results of the Swedish Doppler-echocardiographic study (SWEDIC). *Eur J Heart Fail.* 2004;6:453-461.

18. Takeda Y, Fukutomi T, Suzuki S, Yamamoto K, Ogata M. Effects of carvedilol on plasma B-type natriuretic peptide concentration and symptoms in patients with heart failure and preserved ejection fraction. *Am J Cardiol.* 2004;94:448-453.

19. van Veldhuisen DJ, Cohen-Solal A, Böhm M, Anker SD, Babalis D. Beta-blockade with nebivolol in elderly heart failure patients with impaired and preserved left ventricular ejection fraction: Data From SENIORS (Study of Effects of Nebivolol Intervention on Outcomes and Rehospitalization in Seniors With Heart Failure). *J Am Coll Cardiol.* 2009;53:2150-2158.

© 2022 Xiang B et al. *JAMA Network Open.*
